# Capsular closure in patients with femoroacetabular impingement syndrome (FAIS): results of a matched-cohort study from the Danish hip arthroscopy registry

**DOI:** 10.1093/jhps/hnaa033

**Published:** 2020-10-07

**Authors:** Bjarne Mygind-Klavsen, Bent Lund, Torsten Grønbech Nielsen, Otto Kraemer, Per Hölmich, Niels Maagaard, Søren Winge, Martin Lind

**Affiliations:** Department of Orthopedics, Section of Sports Traumatology, Aarhus University Hospital, Aarhus N 8200, Denmark; Department of Orthopedics, Horsens Regional Hospital, Horsens 8700, Denmark; Department of Orthopedics, Section of Sports Traumatology, Aarhus University Hospital, Aarhus N 8200, Denmark; Department of Orthopedic Surgery, Copenhagen University Hospital, Amager-Hvidovre, Sports Orthopedic Research Center – Copenhagen (SORC-C), Hvidovre 2650, Denmark; Department of Orthopedic Surgery, Copenhagen University Hospital, Amager-Hvidovre, Sports Orthopedic Research Center – Copenhagen (SORC-C), Hvidovre 2650, Denmark; Department of Orthopedics, Odense University Hospital, Odense 5000, Denmark; Department of Orthopedics, CFR Private Hospital, Hellerup 2900, Denmark; Department of Orthopedics, Section of Sports Traumatology, Aarhus University Hospital, Aarhus N 8200, Denmark

## Abstract

Capsular closure in femoroacetabular impingement syndrome (FAIS) patients during hip arthroscopy procedures is debated. The Danish Hip Arthroscopy Registry (DHAR) contains data to perform matched-cohort analyses. The purpose of this study is to evaluate 1-year subjective outcome data from DHAR after hip arthroscopy for FAIS with capsular closure and compare these outcome data with a matched-cohort study group. The primary hypothesis was that there would be no difference in Copenhagen Hip and Groin Outcome Score (HAGOS) subjective outcome between patients with and without capsular closure. This is a retrospective cohort study (level of evidence, 3). FAIS patients eligible for hip arthroscopy between January 2012 and December 2017, and where the interportal capsulotomy was closed, were identified and matched with patients without capsular closure. Matching criteria were gender (1:1), age (±5 years), degree of cartilage injury: ICRS and modified Becks grade (±1 grade) and radiological parameters: lateral center edge angle and alpha angle (±10°). A comparison between cohorts regarding differences in patient outcome scores, HAGOS, Hip Sports Activity Scale (HSAS), EuroQol-5 Domain (EQ-5D) and numeric rating scale (NRS) pain at 1-year follow-up were performed. Wilcoxon rank-sum test was used to compare differences between preoperative and postoperative subjective outcome scores, level of statistical significance was 0.05. A total of 189 patients were included in the capsular closure group and matched with 189 control patients. The mean age in years (±standard deviation) was 39.4 (±11.8) and 39.3 (±11.2), respectively, 55% females. Both groups improved significantly at 1-year follow-up. Significant improvements in the capsular closure group were found in HSAS, EQ-5D, NRS pain (rest and walk) and most HAGOS subscales compared with the non-closure group. All patients underwent labral repair in combination with both femoral osteochondroplasty and acetabuloplasty. The revision rate reported after 2 years was 6.8% in the non-closure group and 3.5% in the closure group. One patient in each cohort received a total hip replacement after 2 years. Capsular closure during arthroscopic FAIS treatment resulted in better subjective outcomes and less pain during rest and walking compared with matched controls. Both groups demonstrated improved outcome at 1-year follow-up. Furthermore, capsular closure might result in a lower risk of a revision hip arthroscopy.

## INTRODUCTION

In the recent decade of treating femoroacetabular impingement syndrome (FAIS) [1], the focus has been upon treating not only the impingement morphologies such as pincer and cam but also the involved intraarticular structures such as the labrum, cartilage and synovium. Recently, the hip joint capsule has gained increased scientific interest biomechanically including the questions if, when and how to close the capsule after arthroscopic treatment [2–4].

It has been proposed that capsular closure might result in a more predictable and reliable hip function due to re-establishment of anatomic joint stability leading to a lower hip arthroscopy revision rate [2, 3, 5]. Both biomechanical and clinical considerations about the hip capsule are described in a recently published systematic review by Ortiz-Declet et al. [6]. They concluded that capsular closure is safe and effective in non-arthritic patients undergoing hip arthroscopic procedures and may yield superior short-term outcomes compared with unrepaired capsulotomy and that biomechanical evidence strongly supports the role of capsular repair in maintaining stability of the hip.

Previously, a study from Danish Hip Arthroscopy Registry (DHAR) [7] have demonstrated good outcomes after arthroscopic FAIS surgery. In 10% of these cases, the capsulotomy was closed.

The purpose of the present study was to describe 1-year outcome data from DHAR after FAIS surgery with capsular closure and compare with a matched control group, where the capsulotomy was left unrepaired (non-closure group). Our primary hypothesis was that there were no differences in HAGOS subjective outcome after 1 year in patients undergoing hip arthroscopy with and without capsular closure.

## MATERIALS AND METHODS

The DHAR was initiated in 2012 as a web-based non-obligatory registry that collects subjective outcome data reported by the patient, clinical and surgical information by the surgeon [8] and is approved by the Danish Health Authorities, J.nr. 2012-58-0006. Every surgeon in Denmark, who performs hip arthroscopies, is encouraged to participate and report data to DHAR.

### Patients

FAIS patients in DHAR with interportal capsular closure during the hip arthroscopy between January 2012 and December 2017 were identified. The FAIS patients were identified according to radiological findings such as cam, pincer or mixed-type impingement as described by Ganz et al. [9] . Since no scientific consensus on radiological measures of the FAIS morphologies exists, DHAR uses the following cut-off values; cam (alpha angle >55°, measured on 45° Dunn view or cross-table lateral) and pincer (lateral center edge angle >39° or isolated cross-over sign, measured on standing antero-posterior pelvis view) [10]. These morphologies were combined with clinical findings related to FAIS such as positive FADIR test, decreased range of motion (compared with the contralateral hip) and mechanical symptoms.

**Fig. 1:**
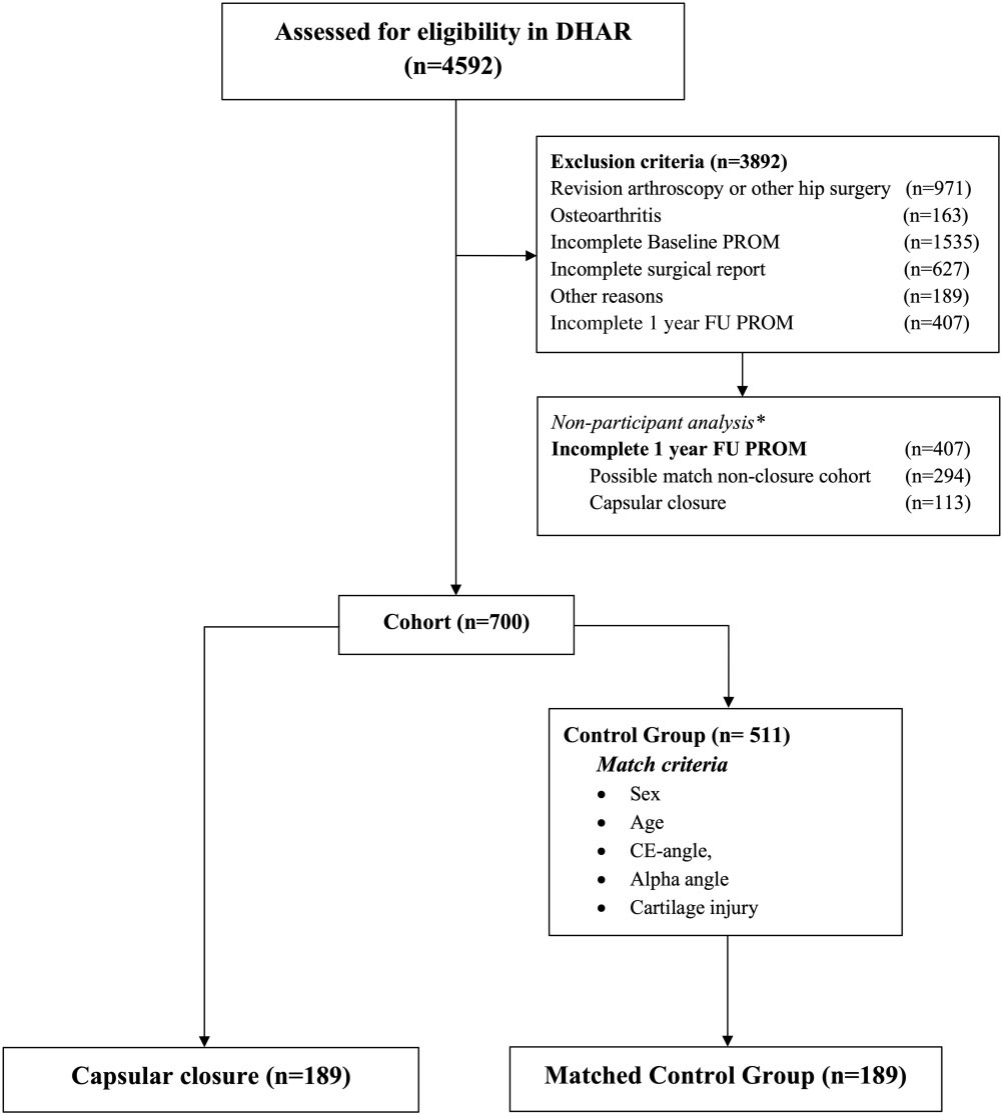
Patient selection for study groups. ^a^Please see comments in the text.

The inclusion criteria were arthroscopic capsular closure performed during FAIS surgery, complete preoperative and 1-year follow-up patient-reported outcome measures (PROM) data, complete surgeon reported data including radiological findings and labral repair. Exclusion criteria were revision hip arthroscopy or other hip-related surgery such as total hip replacement (THR) or periacetabular osteotomy, osteoarthritis (defined as Tönnis grade >1 and/or lateral joint space width <3 mm), labral resection or reconstruction in case of unrepairable labral injury, other hip conditions such as hip dysplasia (defined as CE angle <25° and Tönnis acetabular index angle >10°), Legg-Calvé-Perthes disease, avascular necrosis of the femoral head, radiological signs of possible acetabular retroversion, such as combined prominent ischial spine sign, posterior wall sign and cross-over sign, and incomplete data registration in DHAR. Patient selection is illustrated in Fig. 1. The matched-cohort control group consisted of similar FAIS patients in DHAR where capsular closure was not performed during the FAIS hip arthroscopy procedure and then pair-matched with a capsular closure cohort. The matching criteria were gender (1:1), age (±5 years) and radiological parameters: lateral center edge angle (±10°) and alpha angle (±10°) and degree of cartilage injury according to ICRS (±1 grade) and modified Becks (±1 grade). To evaluate the potential risk of selection bias, a non-participant analysis was performed to assess the potential differences in radiological parameters and PROMs preoperatively between the participants and non-participants within the two cohorts. This was performed to diminish the potential risk of selection bias.

### Outcome evaluation

The PROM used in DHAR included Copenhagen Hip and Groin Outcome Score (HAGOS), The EuroQol-5 Domain (EQ-5D), The Hip Sports Activity Scale (HSAS) and the pain scores used were the numeric rating scale (NRS) at rest and after 15 min of walking. HAGOS is a questionnaire (37 questions in total) aimed for young to middle-aged adults with hip and groin pain and consists of six sub-scales assessing symptoms, pain, function in daily living, function in sport and recreation, participation in physical activities and hip and/or groin-related quality-of-life, each scored separately [11, 12]. EQ-5D is a widely used generic health-related quality-of-life instrument now translated and validated into many languages [13]. HSAS is recommended as a reliable and valid activity measurement useful for patients with FAIS [14]. Pain levels are measured as NRS pain scores at rest and after 15 min of walking (10 being the worst pain score) [15]. These subjective measurements were collected preoperatively and at 1-year follow-up. The radiological data, reported by the surgeon, consist of the following radiological measurements: Wibergs Lateral Centre Edge angle (LCE), lateral joint space width measured at the lateral sourcil, Tönnis acetabular index angle, alpha angle and the presence of posterior wall sign, cross-over sign and prominent ischial spine sign [10]. The surgical technique included interportal capsulotomy, labral repair, cartilage debridement or microfracture, rim trimming (acetabuloplasty) and cam resection (osteochondroplasty) as needed.

Furthermore, patient demographics, such as age and gender, were collected from DHAR as well as information about performed arthroscopic procedures during the hip arthroscopy, describing how the labral and cartilage injuries were handled.

The degree of cartilage injuries is in DHAR classified according to the International Cartilage Repair Society (ICRS) on the femoral side consisting of five grades (Grade 0: normal cartilage, Grade 1: nearly normal, Grade 2: abnormal, Grade 3: partial loss of cartilage and Grade 4: exposed bone) and the modified Becks cartilage classifications [16–18] on the acetabular side consisting of five grades (Grade 0: normal cartilage, Grade 1: fibrillation, Grade 2: wave sign, Grade 3: separation between labrum and articular cartilage and Grade 4: exposed bone).

### Capsular closure surgical technique

All patients, including the patients in the non-closure group, were positioned in the supine position. Standard anterolateral and midanterior portals were established. An interportal capsulotomy between these two portals was performed and placed approximately 10 mm from the labrum and approximately 30 mm in length. At the end of the procedure, in the capsular closure group only, the interportal capsulotomy was closed with No. 2 absorbable Vicryl sutures. A total of 2–3 sutures were placed with approximately 10-mm intervals for a secure side-to-side closure. Surgeons (*n* = 5) treating patients in the capsular closure cohort had in average 37 months (range, 2–56 months) of experience in performing hip arthroscopies and on average performed 272 procedures (range, 6–1018 procedures) before the inclusion of the first patient. The surgeon with 2 month of experience and 6 registered procedures, performed procedures before the initiation of DHAR. Patients in the non-closure cohort were treated by a total of 14 surgeons, including the five surgeons who performed the closures. Their level of experience before the first included patient was on average, 10.1 months (range, 0–38 months) experience in hip arthroscopies and 21 procedures (range, 0–81). The surgeon with ‘zero’ experience and procedures represent the start of DHAR but performed hip arthroscopies before data registration in DHAR started.

### Statistical analysis

Statistical analysis was performed using STATA 15.1 software (StataCorp, College Station, TX, USA). The demographic data were presented as the mean with standard deviation (SD). Student’s *t* test was used to compare differences between the two groups. Chi-square test was used when comparison of binary data is independent. In the treatment of acetabular cartilage damage, three surgical modalities were reported (Table I). In the comparison between the two groups, all surgical modalities were combined in the χ^2^ test. The same method was applied when testing differences in cartilage injury degree and area (Table II). As PROM data were not normally distributed, data were presented as median values and interquartile ranges (25th and 75th percentile). A non-parametric statistic method, Wilcoxon rank-sum test, was used to compare differences between preoperative and postoperative subjective outcome scores. The threshold of statistical significance was set to 0.05 for all analyses.

**Table I.** Comparisons of demographics and major surgical procedures performed between the capsular closure and non-capsular closure cohorts

	Capsular closure (*n* = 189)	Non-capsular closure (*n* = 189)	
	Mean ± SD	Mean ± SD	*P* value
Age (years)	39.4 ± 11.8	39.3 ± 11.2	0.95^*^
Gender, male:female (*n*)	89:100	89:100	
Lateral center edge angle	31.6 ± 4.5	31.7 ± 5.3	0.91^*^
Alpha angle	65.8 ± 13.2	67.4 ± 9.5	0.17^*^
Labrum surgery			
Repair (*n*)	189	189	
Cartilage surgery (*n*)	189	189	
Femoral head chondroplasty (*n*)	0	3	
Femoral head microfracture (*n*)	2	0	
Acetabulum chondroplasty (*n*)	69	87	
Acetabulum microfracture (*n*)	13	6	0.08^**^
Acetabulum debridement (*n*)	105	93	
Femoral head-neck osteochondroplasty (*n*)	189	189	
Acetabuloplasty/rim-trimming (*n*)	189	189	

*P* values indicates comparison between cohorts.

* Students *t* test,

** χ^2^ test comparing the different surgical modalities treating any degree of acetabular cartilage injury within the two cohorts. A *P* values of <0.05 is considered a statistically significant difference.

**Table II.** Distribution of reported cartilage injury grade (ICRS and modified Becks) and estimated area (cm^2^) of injury

	Capsular closure (*n* = 189)	Non-capsular closure (*n* = 189)	*P* value
Femoral head (*n*)			
ICRS 0–1	176	176	0.85*
ICRS 2–4	12	13	
Area = 0 cm^2^	174	167	0.28*
Area < 1 cm^2^	4	10	
Area 1–2 cm^2^	7	6	
Area > 2 cm^2^	3	6	
Acetabulum (*n***)**			
Modified Becks 0–1	30	22	0.28*
Modified Becks 2–4	159	167	
Area = 0 cm^2^	1	1	0.80*
Area < 1 cm^2^	60	53	
Area 1–2 cm^2^	112	122	
Area > 2 cm^2^	15	13	

*P* values (*χ^2^) indicates comparison between cohorts regarding the total difference in cartilage injury grade and area. A *P* value of <0.05 is considered a statistically significant difference.

## RESULTS

A total of 4592 hip arthroscopies were identified in the study period. Hip arthroscopies (*n* = 3892) were excluded due to the defined exclusion criteria. This resulted in 189 FAIS patients who were identified and included in the capsular closure cohort and matched with 189 FAIS patients from DHAR according to the above-mentioned matching criteria. Demographics and major surgical procedures performed during the hip arthroscopy are illustrated in Table I. Table II demonstrates the cartilage injury severity and the estimated damaged area either on the femoral head or in the acetabulum.

There were no significant differences between the cohorts for any of the matching criteria. Fifty-five percent of the patients were females. The mean age in years (±SD) was 39.4 (±11.8) in capsular closure cohort and 39.3 (±11.2) in the matched cohort. The rates of revision hip arthroscopy measured 2 years after the primary hip arthroscopy procedure were, respectively, 6.8% (data available from 177 patients) in the non-closure group and 3.5% (data available from 143 patients) in the closure group. Indications for revision hip arthroscopies in the non-capsular closure cohort were, respectively (indication and percent): adhesions (5%), labral tear (3%), residual impingement (1%), psoastendinopathy (tenotomy) (1%) and new cartilage injury (0.6%). The indications for revision hip arthroscopies in the capsular closure cohort were, respectively (indication and percent): Residual impingement and labral tear (3%) and unknown (0.7%). The relative risk of receiving a revision hip arthroscopy in the non-closure group was 1.94 compared with the relative risk in the capsular closure group.

After 1 year, no patients had received a THR. One patient in each cohort reported conversion to THR after 2 years. These data were extracted from DHAR, based on a total of 217 patient self-reports from the two cohorts.

### Patient-reported outcomes

Individual PROM scores within cohorts and comparisons of PROM scores in the two cohorts are illustrated in Table III. The preoperative PROM scores demonstrated no significant difference [except HAGOS subscale Physical Activity (PA)] between the two groups. In both cohorts, all PROM scores demonstrated statistically significant improvements from preoperative to 1-year follow-up.

**Table III.** Comparison of outcome scores between patients with or without capsular closure presented as median values with interquartile ranges, since data were not normally distributed

	Capsular closure (*n* = 189)	Noncapsular closure (*n* = 189)		*P* value
	Median (25th–75th)	Median (25th–75th)		
HAGOS				
Pain				
Preoperative	55.0 (40.0–67.5)	55.0 (40.0–67.5)	0.65	
1-year postop	85.0 (67.5–95.0)*	72.5 (55.0–87.5)*	<0.01	
Symptoms				
Preoperative	57.1 (42.9–67.9)	53.6 (39.3–67.9)	0.18	
1-year postop	78.6 (64.3–89.3)*	67.9 (53.6–82.1)*	<0.01	
ADL				
Preoperative	60.0 (40.0–75.0)	60.0 (35.0–70.0)	0.36	
1-year postop	90.0 (75.0–95.0)*	80.0 (55.0–90.0)*	<0.01	
Sports				
Preoperative	40.6 (21.9–56.3)	34.4 (18.8–53.1)	0.35	
1-year postop	75.0 (53.1–87.5)*	56.3 (31.3–81.3)*	<0.01	
PA				
Preoperative	12.5 (0.0–25.0)	12.5 (0.0–37.5)	0.01	
1-year postop	50.0 (12.5–87.5)*	37.5 (12.5–75.0)*	0.15	
QOL				
Preoperative	30.0 (20.0–40.0)	30.0 (20.0–40.0)	0.11	
1-year postop	60.0 (40.0–85.0)*	45.0 (30.0–75.0)*	<0.01	
HSAS				
Preoperative	2 (1–3)	2 (1–3)	0.66	
1-year postop	3 (2–4)*	2 (2–4)*	0.02	
NRS rest				
Preoperative	32 (16–52)	34 (15–57)	0.43	
1-year postop	5 (1–21)*	11 (1–26)*	0.04	
NRS walk				
Preoperative	45 (24–68)	50 (26–71)	0.24	
1-year postop	9 (1–26)*	19 (3–48)*	<0.01	
EQ-5D				
Preoperative	0.72 (0.63–0.78)	0.72 (0.66–0.78)	0.69	
1-year postop	0.82 (0.72–1.00)*	0.78 (0.72–0.82)*	<0.01	

* Significant improvement within cohorts between preoperative and 1-year follow-up scores. *P* values indicates differences when comparing between the two cohorts were calculated. A *P* value of <0.05 is considered significant.

When comparing the two cohorts at 1-year follow-up, significantly higher scores in the capsular closure cohort was found in HSAS, NRS, EQ-5D and in all HAGOS subscales, except the PA subscale.

### Non-participant analysis

In the group of possible matches 294 patients, and in the group with capsular closure, 113 patients were excluded before the matching process because of incomplete 1-year follow-up data. An analysis of these non-participants demonstrated no differences in preoperative PROMs compared with the participants. In the capsular closure patients, the analysis demonstrated a significant higher proportion of isolated radiological ischial spine sign in the non-participant group of the capsular closure cohort (*P* = 0.02). In non-capsular closure patients, the non-participant group demonstrated significant higher alpha angles (*P* < 0.01), a significant higher proportion of isolated radiological ischial spine sign (*P* < 0.01) and significant higher degrees of cartilage injury compared with the participants [modified Becks (*P* = 0.02), ICRS (*P* < 0.01)].

## DISCUSSION

The primary finding of this registry-based cohort study was the significantly better patient-reported outcome scores when the capsulotomy was closed in connection with hip arthroscopy for FAIS as measured by HAGOS (except PA subscale), HSAS, NRS pain during rest and walking and EQ-5D. Also, both groups significantly increased the scores in all PROMS at 1-year follow-up. Furthermore, without closure of the capsule, the study demonstrated a higher relative risk of receiving a revision hip arthroscopy within the first 2 years after the primary hip arthroscopy procedure. Revision data after 1 year was very low, consisting of two revision hip arthroscopies in the non-closure cohort and one in the capsular closure cohort. Therefore, 2-year data were included in this study.

In recent years, several factors contributing to the hip stability, including the hip capsule, have been studied intensely and its importance for the stability and function of the hip has been recognized based on cadaveric studies. When gaining access to the hip joint during hip arthroscopy, the usual capsulotomy performed is between the anterior or mid-anterior portal and the anterolateral portal. This capsulotomy might involve parts of the iliofemoral ligament and thereby increase the risk of increased external rotation and anterior translation if not repaired during hip arthroscopy [19]. A study of the distractive strength of the hip fluid seal demonstrated that intact labrum had the primary role in the suction effect at smaller hip joint displacements (1–2 mm) but continued resisting distraction until >6 mm, after which the capsule was the main stabilizer [20]. Furthermore, the effect of acetabular labrum preservation might improve the native hip stability compared to partial or complete excision of the acetabular labrum [21]. As a rare complication to hip arthroscopy, a non-repaired hip capsule might result in iatrogenic hip instability or traumatic hip dislocation during hip extension and external rotation [22]. Therefore, it seems reasonable that repair of the capsule might be important to restore the capsular anatomy at the end of a hip arthroscopy case to prevent instability. When investigating the importance of capsular closure, it seems important to apply more than one suture to approximate the edges of the capsulotomy as seen in the biomechanical study by Chahla *et al*. [4].

Currently, only one retrospective cohort study by Frank *et al*. [23] has presented data from FAIS patients undergoing hip arthroscopy comparing outcomes after complete repair versus partial repair of the T-capsulotomy. That study included 64 FAIS patients and demonstrated that patients undergoing a complete repair of a T-capsulotomy had a significantly higher sport-specific outcome and reported higher levels of satisfaction at final follow-up 2 years after their hip arthroscopy, than patients who only had a partial repair of the T-capsulotomy. They also demonstrated a higher revision rate in the partial repair group due to persistent hip pain and capsular defects identified on magnetic resonance arthrography. These results might differ from the current study since the T-capsulotomy involves a larger opening of the capsule compared with the interportal capsulotomy, which is more often used by the surgeons according to the recently published systematic review by Riff *et al*. [24]. One possible advantage of the T-capsulotomy might be enhanced visibility of the cam morphology due to the larger opening of the capsule and therefore might improve femoral osteochondroplasty procedure, resulting in decreased revision rates. Other studies have investigated the influence of capsular management in hip arthroscopic procedures, but the cohorts are either borderline dysplastic patients [25, 26] or unspecific groups undergoing arthroscopic hip preservation surgery [27]. A study [28] presented retrospectively mid-term follow-up results after capsular closure versus unrepaired capsulotomy with no significant differences between the groups regarding PROMs but suggested that routine capsular closure might lead to a more durable outcome and that patients who had unrepaired capsules had deterioration in mHHS as well as a higher rate of conversion to arthroplasty. Their study did not only include FAIS patients, since patients with a low LCE-angle (CE ≤18°) were included. Furthermore, capsular closure included both plication and repair of the capsulotomy. Therefore, it is difficult to compare the results of the current study with recent presented studies since the study cohorts are not identical.

Despite some literature suggesting clinical benefit of capsular closure the procedure is not generally performed as standard. Gupta *et al*. [29] reported on preferences in surgical procedures in a survey from 27 high-volume orthopedic surgeons specializing in hip arthroscopy. They found that all surgeons performed routine capsulotomy but demonstrated a great variability in how and when to close the capsule. Three surgeons closed the capsule every time. Ten and eleven surgeons, respectively, closed the capsule in more than 50% and less than 50% of the time. Three surgeons did never close the capsule.

Although, the importance of capsular management in FAIS patients is still debatable and future studies are needed to define if the procedure should be performed routinely, the present study suggests that some clinical benefit of capsular closure after hip arthroscopy probably exists and thereby, contribute positively to the surgical management of FAIS patients.

### Limitations

This study revealed various limitations. First, this study included only patients with a complete 1-year follow-up and therefore some patients in both groups were excluded in the patient selection process resulting in possible selection bias. In the group of possible matches 294 patients and in the capsular closure group 113 patients were excluded before matching due to incomplete 1-year follow-up. This illustrates one of the major challenges with registry studies, which is completeness of data. However, an analysis of these non-participants demonstrated no differences in preoperative PROMs but demonstrated minor radiological differences (lower alpha angle and higher proportions of isolated ischial spine sign) and intraarticular pathologies (lower degrees of cartilage injury) when compared with the participants. Second, the capsular closure cohort could only be matched due to the strict matching criteria. Many other criteria could have been selected as possible confounders such as body mass index etc. To limit the effect of this, a randomized controlled trial is needed. Furthermore, matching criteria did not include surgeon or the level of skills. That might also be a possible confounder in this study since it could affect the surgical technique and as a result of that also the outcome. Third, data regarding revision surgery consisting of conversion to THR were based on patient-reported data and was not confirmed with The Danish Health Data Authority. Two-year follow-up revision data were included in the study, since only a few patients received a revision surgery in the study period. Fourth, the indications for closure of the capsulotomy have not been described. Whether the surgeon performed closure in high-level sports participants only or in patients with capsular laxity cannot be detected in DHAR. Recently, capsular closure has gained high scientific interest and might have resulted in a surgical trend rather than a patient specific procedure. Fifth, the length and site of the capsulotomy or used capsular closure technique might vary between surgeons and was not recorded in DHAR. If these data were available, some level of interobserver variability would be expected. Sixth, postoperative rehabilitation in the two cohorts might be different and therefore influence the outcome. Seventh, this study analyzes 1-year follow-up only and therefore it is necessary to test these results in a longer time frame as well as calculating the minimal clinical important difference.

Due to these limitations, a Danish designed multicenter randomized controlled trial was initiated in 2017 (Clinicaltrials.org: NCT03158454) [30].

## CONCLUSION

Capsular closure during arthroscopic FAIS treatment resulted in better subjective outcomes in most HAGOS subscales (except PA), HSAS, EQ-5D and in less pain during rest and walking compared to a cohort without capsular closure. Both groups significantly increased the score in all PROMS at 1-year follow-up. Furthermore, capsular closure resulted in a lower risk of a revision hip arthroscopy. 

## CONFLICT OF INTEREST STATEMENT

None declared.
